# Maintenance of Leukemia-Initiating Cells Is Regulated by the CDK Inhibitor Inca1

**DOI:** 10.1371/journal.pone.0115578

**Published:** 2014-12-19

**Authors:** Nicole Bäumer, Sebastian Bäumer, Frank Berkenfeld, Martin Stehling, Gabriele Köhler, Wolfgang E. Berdel, Carsten Müller-Tidow, Petra Tschanter

**Affiliations:** 1 Department of Medicine A, Hematology/Oncology, University of Muenster, 48129 Muenster, Germany; 2 Interdisciplinary Center for Clinical Research (IZKF), University of Muenster, 48129 Muenster, Germany; 3 Max-Planck-Institute for Molecular Biomedicine, Department of Tissue Morphogenesis, and University of Muenster, Faculty of Medicine, Muenster, 48129 Muenster, Germany; 4 Dept. of Medicine IV, Hematology and Oncology, University of Halle, 06120 Halle, Germany; 5 Institute for Pathology, Clinical Center Fulda, 36043 Fulda, Germany; French Blood Institute, France

## Abstract

Functional differences between healthy progenitor and cancer initiating cells may provide unique opportunities for targeted therapy approaches. Hematopoietic stem cells are tightly controlled by a network of CDK inhibitors that govern proliferation and prevent stem cell exhaustion. Loss of *Inca1* led to an increased number of short-term hematopoietic stem cells in older mice, but Inca1 seems largely dispensable for normal hematopoiesis. On the other hand, *Inca1*-deficiency enhanced cell cycling upon cytotoxic stress and accelerated bone marrow exhaustion. Moreover, AML1-ETO9a-induced proliferation was not sustained in *Inca1*-deficient cells *in vivo*. As a consequence, leukemia induction and leukemia maintenance were severely impaired in *Inca1^−/−^* bone marrow cells. The re-initiation of leukemia was also significantly inhibited in absence of *Inca1^−/−^* in MLL—AF9- and c-myc/BCL2-positive leukemia mouse models. These findings indicate distinct functional properties of Inca1 in normal hematopoietic cells compared to leukemia initiating cells. Such functional differences might be used to design specific therapy approaches in leukemia.

## Introduction

Hematopoietic stem cells (HSCs) are characterized by their ability to self-renew and to differentiate into all hematopoietic lineages. Division and expansion of HSCs have to be tightly regulated to avoid exhaustion but at the same time to ensure sufficient proliferation for maintaining the blood system. Moreover, HSCs and hematopoietic progenitor cells (HPCs) have to be activated in preparation of a stem cell donation for transplantation and intrinsically after injury of the bone marrow i.e. as a consequence of a disease or of chemotherapy.

Remarkably, stem cell expansion is highly sensitive to aberrations of cell cycle regulation. Several CDK inhibitors restrict HSC proliferation [1]–[5]. However, several key cell cycle regulators, such as CDK2 and RB, were shown to be dispensable for stem cell regulation [6]–[8]. For some of the CDK inhibitors, loss-of-function mouse models revealed distinct functions in HSC. Loss of p21 has a strain-specific effect on HSC numbers and proliferation, suggesting that p21 maintains HSC quiescence [2], [9]. A similar function was identified for p27, but at the level of more committed progenitor cells [1]. In this family, especially p57 turned out to be essential for HSC maintenance and self-renewal in recent studies [10], [11]. The absence of p16 attenuated HSC repopulation defects and apoptosis caused by senescence [3]. Deletion of the early G1-phase CDKI p18 resulted in improved long-term engraftment and increased self-renewal of primitive hematopoietic cells [4], [5].

Therefore, different CDKIs have highly specific effects on the regulation of hematopoietic stem cells, possibly because of their indispensable role during cell cycle progression. The complex network of cell cycle regulation encompasses a high degree of compensatory features in most cell types [8], [11]. As a consequence, genetic deletion of CDK inhibitors mainly leads to stem cell specific phenotypes where especially tight cell cycle control is required.

Leukemic stem cells (LSCs) are characterized by the ability to generate leukemic blast cell populations, regardless whether they are made of rare stem cells or are more frequent progenitor cells. Often, leukemia initiating cells are chemoresistant due to their infrequent divisions, which appears to prevent their efficient eradication [12], [13]. Remarkably, it has been investigated that cell cycle restriction due to p21^CIP1^ expression in LSCs is necessary to induce and maintain PML-RARα- or AML1-ETO-driven leukemogenesis in mice [14]. Moreover, the induction of cycling in leukemia stem cells by G-CSF increased their responsiveness to chemotherapy [13]. Still, little is known whether the mechanisms of stem cell pool regulation differ between normal hematopoietic stem cells and leukemic stem cells.

Recently, we identified INCA1 (Inhibitor of CDK interacting with cyclin A1) as a novel interaction partner of cyclin A1/CDK2 [15], [16]. Inca1 binds to CDK2 and acts as an inhibitor of CDK2 similar to p21 and p27. Decreased INCA1 levels in blasts from Acute Lymphoid Leukemia (ALL) and Acute Myeloid Leukemia (AML) patients underlined its relevance for growth control *in vivo* and for the hematopoietic system [15]. Although *Inca1*-knockout mice are viable and fertile, we identified a different spleen architecture in absence of Inca1 [15], possibly hinting at role of Inca1 in normal hematopoiesis. We also discovered that the tumor suppressor Ing5 interacts with and depends on Inca1 [17], further underlining a putative role of Inca1 in cancerogenesis [18].

We used different transduction/transplantation mouse models to investigate the role of Inca1 in leukemogenesis. Bone marrow cells were retrovirally transduced with the respective oncogenes and transplanted into recipient mice. One of the most common genetic abnormalities in acute myeloid leukemia (AML) is the t(8;21)(q22;q22) translocation that results in the fusion protein AML1-ETO. Since the expression of full length AML1-ETO does not lead to the development of leukemia [19]–[21], we took advantage of an alternatively spliced isoform of the AML1-ETO transcript, AML1-ETO9a, which induces an acute myeloid leukemia in mice with a high penetrance [21]. In addition, we used the oncogenes MLL-AF9 that occurs in typically in the FAB-M4 or M5 subtypes of human AML and reliably and rapidly induces an AML in a transduction/transplantion mouse model [22], [23]. Moreover, the co-expression of c-myc and Bcl2 induces a bilinear myeloid–B lymphoid leukemia and can therefore reveal influences on lineage choice in leukemogenesis [24].

Here, we investigated the role of Inca1 in murine normal hematopoiesis and under stress conditions. We show that absence of Inca1 mildly affects normal hematopoiesis under homeostatic conditions but controls hematopoiesis after induction of cytotoxic stress and plays a role in the maintenance of leukemia development in acute myeloid leukemia.

## Materials and Methods

### Animal experiments

*Inca1*-knockout (-/-) mice were generated and genotyped as previously described [14]. All animal experiments in this study were carried out in strict accordance with the recommendations of the institutional animal care and use committee ‘‘Landesamt fuer Natur, Umwelt und Verbraucherschutz NRW”. This study was approved by the institutional animal care and use committee and of the local veterinary administration of Muenster (G15/2005, 8.87-51.04.20.09.322, and 87-51.04.2011.A005). Mice were kept in individually ventilated (IVC-) Typ II cages (Tecniplast GmbH, Germany) in groups of five mice, in a 12-hour light/dark cycle, with room temperature at 22±2°C and a relative air humidity of 45–65%. All mice were allowed free access to water and a maintenance sterile diet. All reasonable efforts were made to ameliorate suffering, including anesthesia using isoflurane inhalation for retro-orbital puncture and isolation of affected mice. Mice were monitored daily for signs of pain or distress. Moribund mice were humanely sacrificed as described below. Study design and biometric planning of each experiment was performed in accordance with a biostatistician. Mice were sacrificed for sample preparation by cervical dislocation after anesthesia. For each experiment, the single animal was an experimental unit.

### Flow cytometry, RNA isolation, real-time quantitative RT-PCR and hematological analysis

Bone marrow, spleen and blood cells were red cell-lysed using AKC-lysis buffer (0.15% NH4Cl, 0.1 M EDTA, 1 mM KHCO_3_, pH 7.4) for 5 min at RT and incubated with the respective antibody (c-Kit, B220, GR1, CD11b, Ter119, CD41, CD45.1, CD45.2, sca-1, CD34, all BD Biosciences, Franklin Lakes, NJ, USA) for 30 min on ice in the dark. Lineage depletion was obtained using the Lineage Cell Depletion Kit Mouse (Miltenyi Biotec, Bergisch Gladbach, Germany) according to the manufacturer's protocol. For analysis of LSK cells, red blood cell-lysed BM and spleen cells were stained with tricolor-conjugated rat antibodies specific for the following lineage markers: CD3, CD4, CD8a, CD45R (B220), and Ly-6G (Gr-1; Invitrogen), and CD11b and Ter119 (eBioscience). LSK cells were sorted using CD117 (c-kit)–allophycocyanin, Sca-1 (Ly-6A/E)–biotin, antistreptavidinphycoerythrin-Cy7, (BD Biosciences) antibodies. LT-HSCs, short-term (ST)–HSCs, and multipotent progenitors (MPPs) were identified using CD34 and CD135 (Flt3) antibodies in addition (BD Biosciences). FACS analysis and sorting of antibody-stained cells [15] and HSC FACS [25], [26] were performed as described previously.

RNA isolation from sorted murine cells was performed using RNeasy Micro Kit (Qiagen, Hilden, Germany) according to the manufacturer's protocol.

Reverse transcription and real-time quantitative RT-PCR were performed as described [16]. The probes were labeled at the 5' end with the fluorescent dye FAM (mInca1, Mm01243673_m1, Life Technologies, Darmstadt, Germany) or VIC (GAPDH) [16] and at the 3' end with the quencher TAMRA.

For concomitant cell cycle analysis in LSK cells, DAPI staining was performed as described [27]. Briefly, antibody-labelled bone marrow cell populations were fixed in 1% formaldehyde for 30 minutes and permeabilized with 0.1% Triton X-100 for 30 minutes then labelled with DAPI (5 µg/ml).

The WBC and blood parameters were analyzed by using the HEMAVET multispecies hematology analyzer (Drew Scientific, UK) following the manufacturer's instruction.

### Colony Formation Assays

Colony formation assays from total primary bone marrow were carried out essentially as described by growing 10,000 red-cell-lysed bone marrow cells per ml methylcellulose with recombinant cytokines for mouse cells including fetal bovine serum, bovine serum albumin, rh insulin, human transferrin (iron-saturated), 2-mercaptoethanol, rm stem cell factor, rm IL-3, rh IL-6, rh erythropoietin in IMDM (MethoCult GF M3434, Stem Cell Technologies) for 7-8 days [26]. Colonies were replated by resuspension of the grown colonies in PBS to test for their self-renewal capacity and by reseeding 10,000 cells per ml methylcellulose. Colony-forming units (CFU) of granulocytes (CFU-G), macrophages (CFU-M), mixed granulocytes/macrophages (CFU-GM) and of erythrocytes (burst-forming units BFU-E/CFU-E) were counted between days 8 and 11.

### Competitive and serial transplantations

Red blood cell-lysed bone marrow of *Inca1^+/+^* and *Inca1^−/−^* mice (CD45.2^+^ C57BL/6N-strain) was mixed 1∶100 ( = 1%), 1∶10 ( = 10%) and 1∶1 ( = 50%) with bone marrow of congenic CD45.1^+^ B6.SJL-mice and a total of one million nucleated cells were injected intravenously into CD45.1^+^ recipient mice, which had been irradiated with 10 Gy. Blood parameters including FACS for the distribution of CD45.1^+^ versus CD45.2^+^ cells (antibodies from BD Biosciences) were analysed at 5 and 12 weeks after transplantation.

For the serial transplantation, bone marrow cells were isolated from 4 age-matched pairs of *Inca1^+/+^* and *Inca1^−/−^* mice. One million nucleated cells that were CD45.2^+^ were transplanted into lethally (10 Gy) irradiated CD45.1^+^ B6.SJL-recipients (three for each donor mouse in each transplantation, without pooling the donor bone marrow cells). Recipients of the first transplantation were sacrificed after 6 weeks and one million CD45^+^ FACS-sorted bone marrow cells were transplanted as described above. This procedure was repeated for another two rounds. Blood, bone marrow, and spleen parameters including FACS for CD45.2^+^ cells were analysed at the day of retransplantation.

### 5-FU exposure *in vivo*

The antimetabolite 5-fluorouracil (5-FU) was used to deplete cycling cells *in vivo*. 5-FU was administered intraperitoneally (i. p.) weekly at a dose of 150 mg/kg body weight, and the survival rate of each group was defined.

For cell cycle analysis, mice were injected on day 0 with the full dose and on day 8 with the half dose of 5-FU to ensure survival of the animals until the examination on day 11. BrdU was injected at 100 µg/g body weight i. p. 10 hrs before preparation. Cell cycle analysis was performed using BrdU/propidium iodide (PI) staining as described previously [28], apoptosis rates were determined using an Annexin-V-FITC kit according to the manufacturer's recommendations (Beckman-Coulter, Krefeld, Germany).

### Histology

Histological sections and hematoxyline-eosine staining of paraffin-embedded tissues were performed according to standard procedures.

### Retroviral transduction, colony assays, and cloning efficiency assays

The plasmids MSCV-AML1-ETO9a-IRES-GFP and MSCV-MLL-AF9-IRES-GFP were kind gifts of Dong-Er Zhang and Frank Rosenbauer, respectively [21], [22]. For the MSCV-c-myc/BCL2-IRES-mCherry, the vector MSCV2.2-cmyc-IRES-BCL2 (from Frank Rosenbauer; [22]) was linearized using ClaI and blunt-ended and a blunt-ended IRES-Cherry from LeGO-iC (http://www.addgene.org/27362/) was ligated into this vector.

The retroviral transduction of lin- bone marrow cells with oncogene-containing viral particles was performed as described previously. Retroviral supernatants were collected as described [15]. For transduction, viruses were bound to retronectin-coated plates by centrifugation as described [29]. Briefly, lineage-depleted bone marrow cells were stimulated overnight, transduced by growth on the virus-coated plates for 24 h and sorted by FACS for EGFP-positivity.

For colony assays, 1,000 EGFP-positive freshly transduced cells per ml methylcellulose M3434 (Stem Cell Technologies) were plated. The total number of GFP-positive colonies was determined on day 10 after plating.

For colony formation assays of transplanted mice, bone marrow cells were isolated and GFP-positive and lineage-negative (AML1-ETO9a) or c-kit-positive (MLL-AF9) cells were sorted by using the FACSAria cell sorter (BD Bioscience, San Jose, CA, USA). Initially, 1,000 cells per ml methylcellulose of were seeded. Subsequently, 10,000 cells/ml methylcellulose were serially replated at a 7 day interval.

To determine the cloning efficiency of bone marrow cells, different concentrations (1, 10, 30, 100 and 300) of FACS-sorted GFP-positive and lineage-negative (AML1-ETO9a) or c-kit-positive (MLL-AF9) cells were seeded in 200 µl methylcellulose in 14 wells of a 48-well plate. 7 days later wells with one or more colonies were classified as positive. The colony-forming unit frequency was determined by Poisson statistical analysis (L-calc software, Stem Cell Technologies).

### Transplantations

Bone marrow cells of wild type and *Inca1*-knockout recipients were retrovirally transduced as described above. Unsorted 100,000 or 250,000 (AML1-ETO9a) or 100,000 (MLL-AF9) GFP-positive cells were transplanted by tail-vein injection into C57Bl/6N wild type recipients, which were lethally irradiated with 8 Gy. Cells transduced with the c-myc/BCL2-retrovirus were sorted for mCherry expression and 50,000 mCherry-positive cells were transplanted along with 10^6^ freshly isolated B6.SJL bone marrow cells to ensure survival of the mice.

For secondary transplantation, bone marrow cells of leukemic mice were isolated and unsorted 2−7×10^5^ AML1-ETO9a-GFP-positive, 10^6^ MLL-AF9-GFP-positive, or 10^6^ c-myc/BCL2-mCherry-positive cells of each individual donor were intravenously injected into irradiated secondary C57Bl/6N wild type mice.

All transplanted mice were dosed with the antibiotic Cotrimoxazol (100 mg/l) (Ratiopharm, Ulm, Germany) until 2 weeks after transplantation to prevent infections during the immunocompromised state after irradiation. The results of the survival experiments were analyzed with the log-rank non-parametric and represented as Kaplan-Meier survival curves. Moribund animals were euthanized by isoflurane inhalation and cervical dislocation. Mice were stated as moribund when they showed certain signs of sickness i.e. shiver, weight loss, neglected grooming.

## Results and Discussion

### The role of Inca1 in the hematopoietic stem cell compartment

We hypothesized that Inca1 as a novel CDK-inhibitor could have impact on cells of the hematopoietic system. This would be in concordance with the finding that several CDKIs have a distinct function in hematopoiesis [1]–[3], [5], [10]. Moreover, we already identified a disturbed architecture of *Inca1*-knockout spleens and expression of Inca1 in hematopoietic and leukemic cells [15]. We therefore further examined Inca1 functions in hematopoiesis.

**Figure 1:**
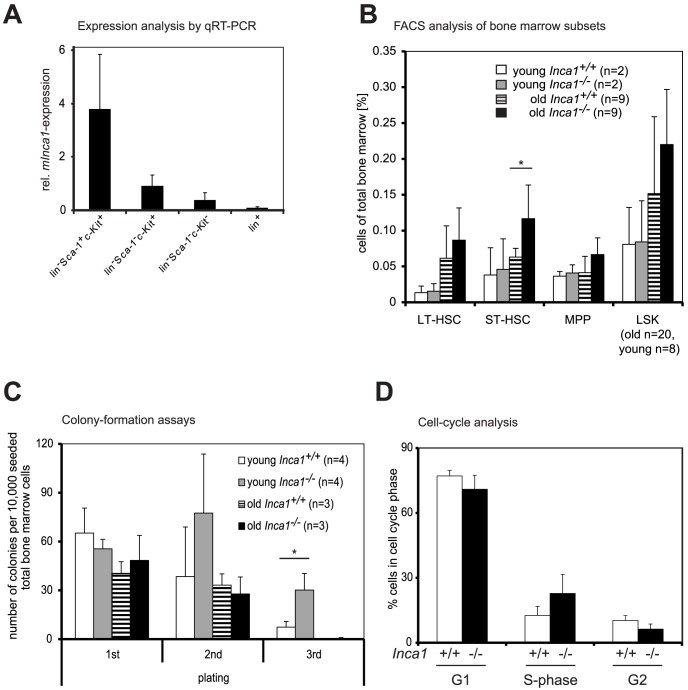
Bone marrow of *Inca1^−/−^* mice contains an enlarged short-term hematopoietic stem cell (ST-HSC) pool. **A.** Inca1 expression was determined by quantitative RT-PCR relative to GAPDH in subpopulations of the bone marrow, which were sorted by FACS. Highest expression was detected in lineage-negative, Sca-1^+^, c-Kit^+^ HSCs (LSK), while expression was lower in primitive lineage-committed progenitors (lin^−^, Sca-1^−^, c-Kit^+^), in more committed progenitors (lin^−^, Sca-1^−^, c-Kit^−^), and in cells expressing lineage-markers (lin^+^). Shown here are means with standard errors from two independent experiments. **B.** The fraction of hematopoietic subpopulations was determined in bone marrow cells from *Inca1^+/+^* and *Inca1^−/−^* mice. FACS analysis revealed that older *Inca1^−/−^* mice (>300 days) had a higher proportion of short-term HSCs (ST-HSC) than their age-matched *Inca1^+/+^* counterparts (p = 0.021, t-test). This effect was not observed in younger mice (<140 days). Indicated are means with standard deviation. **C.** *Inca1^+/+^* and *Inca1^−/−^* bone marrow cells from age-matched mice were analyzed in colony formation assays with subsequent replating. Replated *Inca1^−/−^* cells of young mice exhibited a significant growth advantage in comparison to wild type cells after two platings (asterisks indicate p = 0.02, t-test) without altering colony differentiation ( Fig. S1B). Shown here are means with standard deviations of four (“young”: 10–18 weeks old mice) and three independent experiments (“old”: 20 months old mice). **D.** For cell cycle analysis of enriched hematopoietic stem cells, Lin^−^ Sca-1^+^ c-Kit^+^ (LSK) cells of age-matched *Inca1^+/+^* and *Inca1^−/−^* mice were stained with DAPI.

First, expression of *Inca1* was analyzed in cell populations sorted by flow cytometry from wild type murine bone marrow by real-time quantitative RT-PCR. *Inca1* mRNA was found predominantly in the HSC-enriched population of lineage-negative Sca-1^+^c-Kit^+^ cells (LSK cells; Fig. 1A), while its expression was decreased in more committed progenitor cells (Fig. 1A) as well as in B-cell precursors (B220^+^) but not in myeloid, erythrocytic, or megakaryocytic progenitor cells [15], revealing a distinctive expression pattern of *Inca1* in normal bone marrow.

Inca1 expression especially in the hematopoietic stem/progenitor cell compartment led to the assumption that Inca1 could regulate the proliferation of these cells. Interestingly, FACS analyses of the bone marrow (see S1A Fig. for an example) of older *Inca1*-deficient mice revealed that the short-term stem cell population was significantly increased compared to their wild type littermates (Fig. 1B, p = 0.021), which was not observed in the bone marrow of young mice (Fig. 1B). Other hematopoietic subpopulations were not significantly disturbed in absence of *Inca1*.

To assess proliferation capacity of *Inca1^−/−^* bone marrow cells, we performed serial colony assays, which show the self-renewal capacity of colony-forming cells in each round of replating [30], from whole bone marrow of age-matched mice. After the first plating, no difference was observed in the number of colonies formed from wild type or *Inca1^−/−^* bone marrow cells (Fig. 1C). However, when these colonies were serially replatedthe absence of *Inca1* was associated with a significant increase in the number of colonies compared to wild type bone marrow from young mice (Fig. 1C; p = 0.02), indicating a growth advantage of colony-forming cells in absence of Inca1. Replated cells of old mice did not grow after two platings (Fig. 1C), most likely due to an exhaustion of colony-forming cells, which represent the hematopoietic progenitor pool. Loss of *Inca1* did not alter the differentiation of the colony forming units ( S1B Fig.).

To follow up on an anti-proliferative effect of Inca1 in hematopoietic cells, we analyzed the cell cycle status of the LSK compartment of older *Inca1^−/−^* and wild type mice (older than 17 months) using multi-color FACS analysis with DAPI staining. Remarkably, the results indicated no significant increase of cells in S-phase in the *Inca1^−/−^* mice compared to age-matched controls (Fig. 1D). Also, colony formation of LSK cells of young mice did not differ between *Inca1^+/+^* and *Inca1^−/−^* cells (S1C Fig.).

**Figure 2:**
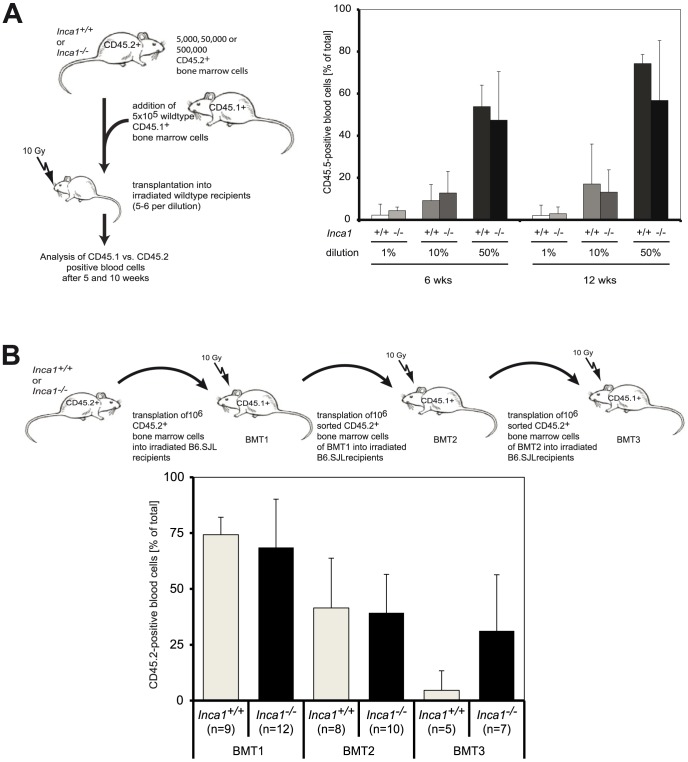
*Inca1* regulates the hematopoietic stem cell pool. **A.** Left-hand side: Schematic overview about the competitive transplantation assays. Cells from CD45.2^+^ *Inca1^+/+^* or *Inca1^−/−^* mice were mixed at different ratios with *Inca1* ^+/+^ bone marrow from congenic CD45.1^+^ mice and transplanted in CD45.1^+^ recipients. Right-hand side: Blood analysis revealed that recipients of both genotypes showed comparable numbers of CD45.2^+^-donor cells in the blood after six and twelve weeks. **B.** For serial bone marrow transplantation, cells from CD45.2^+^ *Inca1^+/+^* or *Inca1^−/−^* mice were transplanted into congenic irradiated CD45.1^+^ mice. Retransplantation was performed six weeks after initial transplantation. Although *Inca1^−/−^* cells appeared overrepresented in the third round of bone marrow transplantation (“BMT3”) compared to *Inca1^+/+^* cells, these results did not reach statistical significance (p = 0.07, t-test).

Next, we analyzed whether Inca1 controlled the proliferation of hematopoietic cells *in vivo*. In competitive transplantation assays, we transplanted bone marrow cells from CD45.2-positive *Inca1* ^+/+^ or *Inca1^−/−^* siblings versus CD45.1-positive bone marrow of congenic mice at ratios of 1∶100 ( = 1%), 1∶10 ( = 10%) and 1∶1 ( = 50%; Fig. 2A, left-hand side), and measured the ratio of CD45.1 vs CD42.2 positive cells by FACS at certain time points after transplantation as a value for the proliferation capacity of *Inca1*-wild type and *Inca1*-knockout cells. Neither five nor 12 weeks after transplantation, *Inca1^−/−^* donor cells possessed a significant advantage compared to *Inca1* ^+/+^ cells (Fig. 2A).

Also, serial transplantations did not exhibit a significant difference between the growth capacity of *Inca1^−/−^* compared to *Inca1^+/+^* bone marrow cells (Fig. 2B), suggesting a rather mild impact of Inca1 on the *in vivo*-function of short-term hematopoietic stem and progenitor cells as detected only in the presence of more ST-HSCs in old *Inca1^−/−^* mice (Fig. 1B). Although some CDK inhibitors like p21 [2], p27 [1] and p16 [3] are important regulators of HSC function, other prominent cell cycle regulators like RB1 [7], CDK2 [6] are largely dispensable for normal hematopoiesis, just as Inca1 seems to be.

### Proliferation of hematopoietic progenitor cells is regulated by Inca1 under 5-FU-induced stress

**Figure 3:**
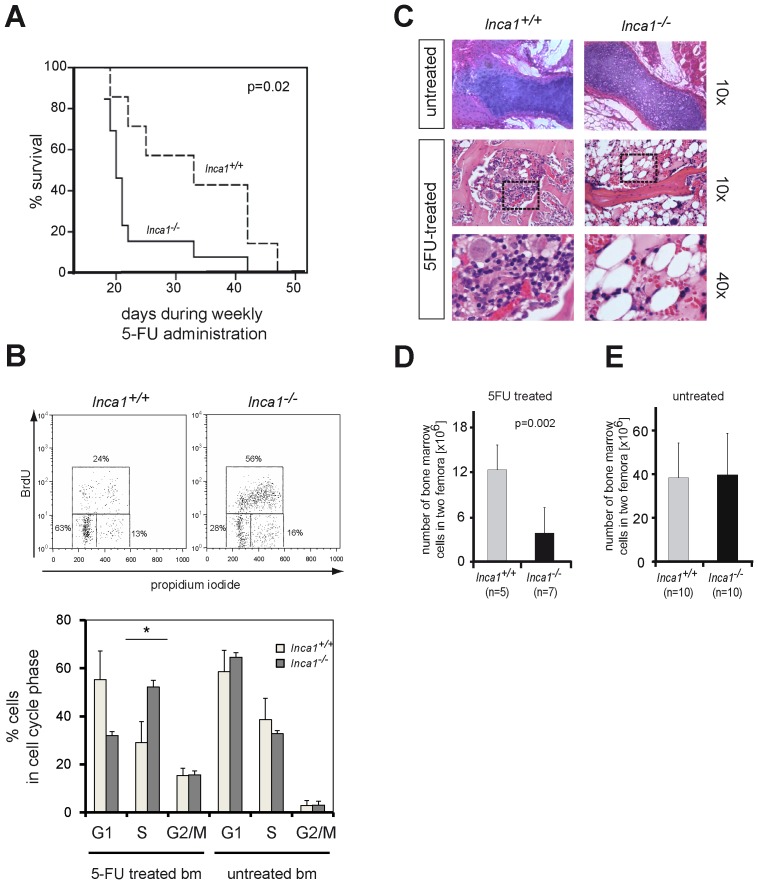
Cytotoxic stress exhausts the stem cell pool in *Inca1*-deficient bone marrow. **A.** The Kaplan-Meier plot illustrates the survival of *Inca1^+/+^* (n = 7) and *Inca1^−/−^* (n = 13) mice after weekly administration of the myeloablative agent 5-fluorouracil (5-FU). 5-FU treatment targeted predominantly proliferating bone marrow cells and led to a significantly shortened lifespan of *Inca1^−/−^* mice compared to their wild type littermates (p = 0.02, log-rank). **B.** Cell cycle analysis of total bone marrow from mice treated twice with 5-FU using BrdU/PI staining and subsequent FACS analysis. *Inca1^−/−^* bone marrow cells of mice that had been exposed twice to 5-FU showed more cells in S-phase than 5-FU treated wild type mice. Shown here is a representative example of three independent experiments. **C.** Histological bone sections from 5-FU treated mice showed significant depletion of hematopoietic cells in the bone marrow of *Inca1^−/−^* sternum (right-hand side) with replacement by fat cells compared to wild type control mice. In contrast, hematopoietic cell numbers were only mildly decreased in wild type mice (left panel). Lower panels show higher magnifications of the areas marked in the upper panels. **D.** The total number of nucleated bone marrow cells was significantly decreased in *Inca1^−/−^* mice after two cycles of 5-FU treatment (mean ± SD, p = 0.002, t-test). All mice were 16 weeks old at the time of analysis. **E.** Bone marrow cellularity in non-challenged mice did not differ between *Inca1^−/−^* and *Inca1^+/+^* genotypes (mice aged 70 to 425 days; mean ± SD, p = 0.83, t-test).

Although Inca1 had a limited impact on steady-state hematopoiesis, it could influence hematopoiesis under stress conditions. Cytotoxic stress specifically affects hematopoietic stem cells by accumulation of potentially detrimental mutations as well as by inducing a strong proliferative signal due to killing of proliferating progenitor cells [31]. We therefore examined the effects of repeated administration of (5-FU). 5-FU ablates cycling cells from murine bone marrow but largely spares quiescent cells, thereby strongly forcing HSCs to divide [14], [15], [32], [33]. Repeated administration of 5-FU eliminates cells that derive from HSCs and hematopoietic progenitor cells (HPC) to replace the ablated cycling cells and subsequently reveals the capacity of HSC/HPCs to enter the cycle until the HSC/HPC pool exhausts [2]. Remarkably, weekly administration of 5-FU significantly shortened the lifespan of *Inca1*-deficient mice compared to wild type littermates (Fig. 3A; p = 0.02). The increased 5-FU toxicity in *Inca1^−/−^* bone marrow suggested a premature exhaustion of the progenitor/stem cell pool most likely reflecting a more rapid cell cycling of HSC/HPC in the absence of *Inca1*.

To confirm this assumption, we injected mice with 5-FU on day 0 and day 7 and performed cell cycle analysis by BrdU incorporation and propidium iodide (PI) staining on day 11. Indeed, *Inca1^−/−^* bone marrow cells of mice that had been exposed twice to 5-FU showed significantly more cells in S-phase than 5-FU treated wild type mice (Fig. 3B, upper and lower panel).

In contrast, the percentage of cells undergoing apoptosis was identical in both 5-FU treated groups in this assay (data not shown). Also, the cell cycle distribution of total bone marrow from untreated *Inca1^−/−^* mice did not differ from wild type littermates (see below, Fig. 3B). Interestingly, bone sections indicated that the bone marrow was largely depleted of hematopoietic cells in 5-FU treated *Inca1^−/−^* mice (Fig. 3C). As a consequence, the numbers of bone marrow cells were significantly decreased in 5-FU treated *Inca1^−/−^* compared to control mice (Fig. 3D; p = 0.002). No other specific histopathological abnormalities were found in the 5-FU exposed *Inca1^−/−^* mice. In addition, the total number of bone marrow cells in untreated *Inca1*-deficient mice was unchanged compared to age-matched wild type mice (Fig. 3E).

Therefore, Inca1 might regulate proliferation of HSC/HPCs under stress conditions, preventing their early exhaustion.

### Inca1 alters latency and penetrance of leukemogenesis induced by AML1-ETO9a

**Figure 4:**
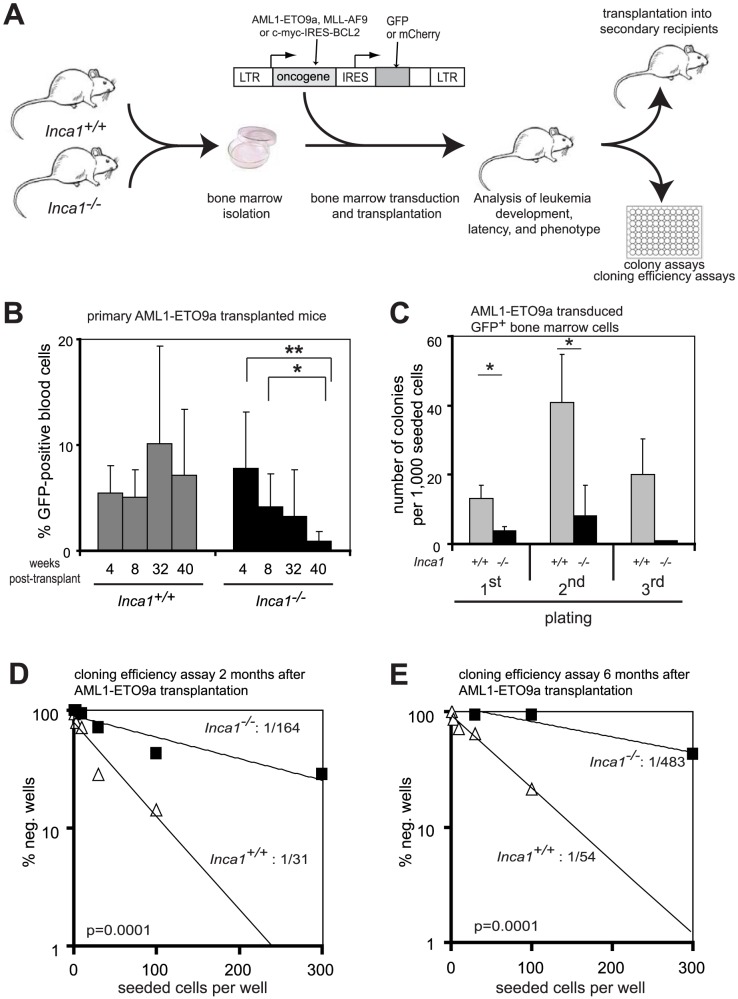
Self-renewal of AML1-ETO9a-transduced bone marrow cells is impaired in absence of *Inca1*. **A.** Schematic overview about the performed transduction and transplantation experiments. Bone marrow isolated from *Inca1^+/+^* or *Inca1^−/−^* mice was retrovirally transduced with AML1-ETO9a-IRES-GFP, MLL-AF9-IRES-GFP, or c-myc-IRES-BCL2-IRES-mCherry. Equal numbers of positive cells were transplanted into lethally irradiated recipients, which were then subjected to different analyses. **B.** Engraftment of AML1-ETO9a-transduced *Inca1^+/+^* and *Inca1^−/−^* bone marrow cells was determined by FACS analysis of GFP-positive cells in the blood of transplanted mice from 4 to 40 weeks. Engraftment was initially higher in recipients transplanted with *Inca1^−/−^* bone marrow cells. The number of GFP-positive cells in *Inca1^−/−^* bone marrow decreased significantly from weeks 4 and 8 until week 40 (*p = 0.015 and **p = 0.04, respectively). This analysis was restricted to mice without overt leukemia. *Inca1^+/+^*: n = 14 at 4 and 8 weeks, n = 9 for 32 and 40 weeks; *Inca1^−/−^*: n = 10 at 4 weeks, n = 9 at 8 and 32 weeks, n = 7 at 40 weeks. **C.** Colony assays with two subsequent replatings using AML1-ETO9a-positive *Inca1^+/+^* or *Inca1^−/−^* bone marrow cells, respectively, that were FACS-sorted from non-leukemic transplanted mice (n = 3 from each genotype). *Inca1^−/−^* bone marrow cells transduced with AML1-ETO9a formed less colonies and replated worse than transduced *Inca1^+/+^* cells (1^st^ plating: p = 0.01; 2^nd^ plating: p = 0.03; 3^rd^ plating: n.s.). **D and E.** For a cloning efficiency assay, 1 to 300 GFP-positive *Inca1^+/+^*; AML1-ETO9a or *Inca1^−/−^*; AML1-ETO9a bone marrow cells from non-leukemic transplanted mice were FACS-sorted two (**D**) or six months (**E**) and lin^−^GFP^+^ cells were seeded in semi-solid medium in a 48-well plate (n = 14 for each concentration). Two months after transplantation, *Inca1^+/+^* cells had a clone forming frequency of 1/31, while the frequency was much lower in *Inca1^−/−^* cells (1/164; p = 0.0001) (**D**). After six months, cloning efficiency of *Inca1^−/−^* cells decreased to 1/483 (**E**).

The sensitivity of *Inca1^−/−^* bone marrow cells towards 5-FU led us to analyze Inca1 functions during another stress situation: leukemogenesis. In leukemia (as in other cancers), initiation and maintenance of the disease depend on exact cell cycle regulation of the leukemia initiating cells that allow unrestricted proliferation while preserving self-renewal activity. We therefore analyzed whether the balance between proliferation and self-renewal was affected by the absence of Inca1. Wild type or *Inca1^−/−^* bone marrow cells were retrovirally transduced with leukemogenic oncogenes and transduced cells were analyzed freshly as well as after primary and secondary transplantations (Fig. 4A).

We first looked at the influence of Inca1 in AML1-ETO9a-driven leukemia [21]. Initial engraftment after primary transplantation appeared to be higher with *Inca1^−/−^* bone marrow as assessed by the detection of GFP positive blood cells, which originated from AML1-ETO9a-transduced bone marrow cells (Fig. 4B). Intriguingly, we observed exhaustion of the transplanted AML1-ETO9a-positive *Inca1^−/−^* bone marrow cells as indicated by the decrease of GFP^+^ peripheral blood cells over time in the *Inca1^−/−^* transplanted mice but not in the mice transplanted with *Inca1^+/+^*; AML1-ETO9a cells (Fig. 4B; p = 0.015 *Inca1^−/−^* at 4 wks vs *Inca1^−/−^* at 40 wks, and p = 0.04 *Inca1^−/−^* at 8 wks vs *Inca1^−/−^* at 40 wks). Also, colony forming units were reduced in *Inca1^−/−^*; AML1-ETO9a cells from mice which were transplanted six months before (Fig. 4C). Replating capability, which was readily observed in *Inca1^+/+^*; AML1-ETO9a bone marrow cells, was abolished in *Inca1^−/−^*; AML1-ETO9a bone marrow cells (Fig. 4C).

We hypothesized that loss of Inca1 led to exhaustion of AML1-ETO9a-transduced stem cells, in analogy to the premature exhaustion of 5FU-treated *Inca1^−/−^* HSC/HPCs. To test this hypothesis, we performed cloning efficiency experiments of GFP^+^ bone marrow cells (as depicted in Fig. 4A) at different time points after transplantation to determine the fraction of AML1-ETO expressing cells that could give rise to clonal growth. AML1-ETO9a-positive cells from transplanted mice were sorted according to their GFP-positivity and seeded in cell numbers from 1 to 300 cells in methylcellulose in 48-well plates and the colony forming efficiency was determined according to Poisson-statistics. GFP-positive *Inca1^+/+^*; AML1-ETO9a exhibited a more than five times higher cloning efficiency than *Inca1^−/−^*; AML1-ETO9a cells at 60 days after transplantation (Fig. 4D). Strikingly, the fraction of GFP^+^ cells that could give rise to clonal growth was strongly diminished in AML1-ETO9a-transduced *Inca1^−/−^* bone marrow cells after six months of repopulation in mice without developing an AML (Fig. 4E).

**Figure 5:**
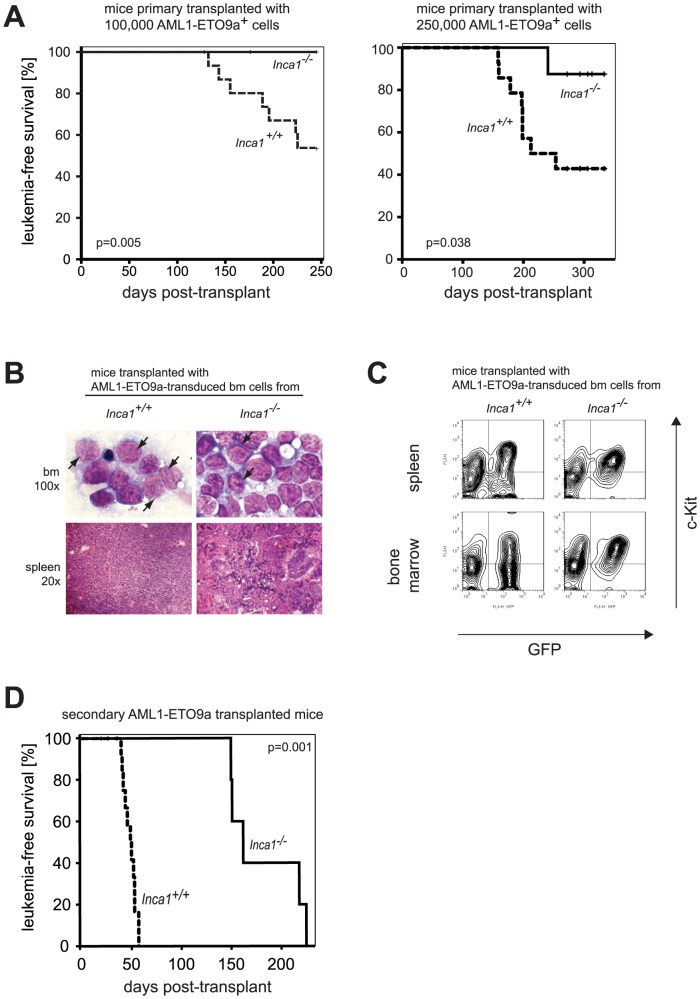
Inca1 is required for AML1-ETO9a-driven leukemia initiation and maintenance *in vivo*. **A.** Survival curve of recipient mice which were transplanted in two independent experiments with bone marrow cells of *Inca1^+/+^* and *Inca1^−/−^* mice that were retrovirally transduced with AML1-ETO9a. Left-hand side: Transplantation with 100,000 AML1-ETO9a^+^ cells (both genotypes: n = 15); right-hand side: Transplantation with 250,000 AML1-ETO9a^+^ cells (*Inca1^+/+^*: n = 14, *Inca1^−/−^*: n = 10). Only one *Inca1^−/−^* bone marrow transplanted with 250,000 AML1-ETO9a^+^ cells led to a lethal leukemic phenotype, while about half of the *Inca1^+/+^* AML1-ETO9a transplanted mice died within 300 days. **B.** Bone marrow smears (upper panel) and spleen sections (lower panel) of *Inca1^+/+^* and *Inca1^−/−^* AML1-ETO9a transplanted mice that died of a leukemic phenotype. Arrows in the upper panels hint at leukemic blasts, indicating acute myeloid leukemia. HE stained sections revealed morphological disruption of the splenic structure and accumulation of myeloid cells in both genotypes (lower panels). **C.** Leukemic phenotypes of recipients that received AML1-ETO9a-transduced *Inca1^+/+^* and *Inca1^−/−^* bone marrow cells are characterized by an infiltration of bone marrow and spleen with GFP^+^c-kit^+^ leukemic blasts in both genotypes. **D.** Survival curve of secondary recipient mice which were transplanted with bone marrow cells of leukemic mice derived from the primary transplantation shown in Fig. 5A. All mice transplanted with primary leukemic *Inca1^+/+^*; AML1-ETO9a bone marrow cells died within 60 days, whereas secondary recipients of *Inca1^−/−^*; AML1-ETO9a cells died with an increased latency of 150 days (*Inca1^+/+^*: n = 18, *Inca1^−/−^*: n = 5; p = 0.001).

In line with these findings, leukemia induction in AML1-ETO9a-transduced *Inca1^−/−^* bone marrow cells was severely impaired (Fig. 5A). We transplanted 100,000 (Fig. 5A, left-hand side) or 250,000 AML1-ETO9a+ cells (Fig. 5A, right-hand side) in two independent experiments. About half of the wild type cell-transplanted recipients died due to acute myeloid leukemia with a latency of about six months in both settings. The leukemic phenotype was characterized by an increased white blood cell count and spleen size (data not shown) and bone marrow and splenic infiltration of c-kit-positive leukemic blasts (Fig. 5B and C). In contrast, mice transplanted with *Inca1^−/−^* cells transduced with AML1-ETO9a showed very low penetrance and a significantly prolonged latency (Fig. 5A). Only one out of ten transplanted mice with 250,000 AML1-ETO9a-transduced *Inca1^−/−^* bone marrow (Fig. 5A, left-hand side) died of acute myeloid leukemia (AML), none out 15 transplanted with 100,000 cells (Fig. 5A, right-hand side). The disease phenotype in this mouse was similar to the phenotype observed in wild type cells (Fig. 5B), with prominent c-kit expression of leukemic blasts (Fig. 5C).

Another assay to analyze the repopulation capacity of leukemic cells is the transplantation of primary leukemic cells into secondary recipients. The leukemia induced in *Inca1^+/+^*; AML1-ETO9a cells was readily transplantable and lethal with very short latency (Fig. 5D). In contrast, mice receiving *Inca1^−/−^*; AML1-ETO9a bone marrow cells of the single leukemic mouse of this genotype (Fig. 5B and C) developed a disease with a significantly delayed latency (more than 150 days; Fig. 5D: p<0.001).

Here, the high proliferative stress induced by leukemogenic oncogenes inhibited leukemia development and maintenance in absence of *Inca1*. Strongly decreased cloning efficiency and colony growth of AML1-ETO9a-expressing bone marrow cells provided functional evidence that Inca1 is required for leukemic stem cell (LSC) maintenance.

**Figure 6:**
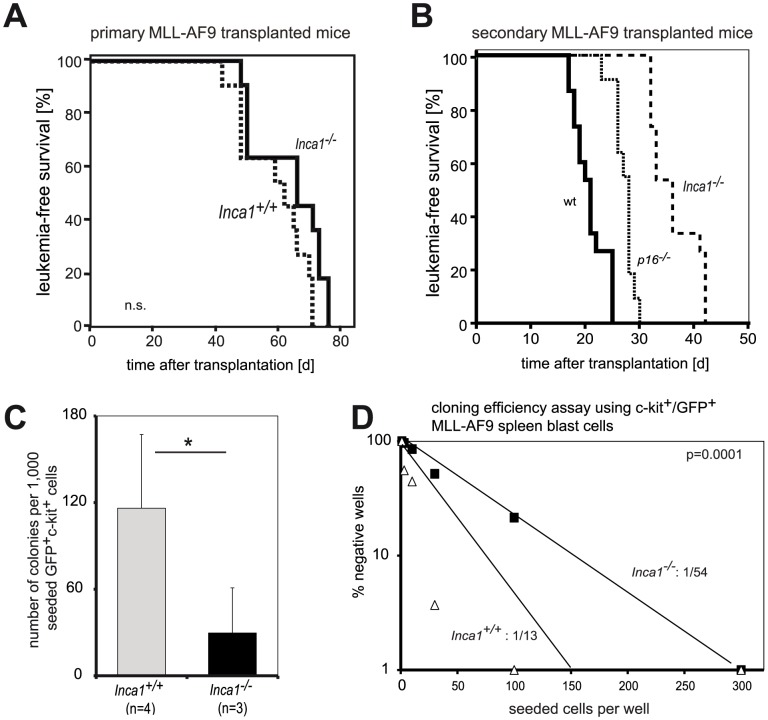
Absence of *Inca1* accelerates MLL-AF9 driven murine leukemogenesis. **A.** Survival curves of recipient mice, which were transplanted with bone marrow cells of *Inca1^+/+^* or *Inca1^−/−^* mice that were retrovirally transduced with MLL-AF9 as depicted in Fig. 4A (n = 11 of each genotype). Cells of both genotypes led to a fatal leukemic disease with comparable latency. **B.** Survival curves of secondary recipient mice which were transplanted with 10^6^ GFP^+^ leukemic spleen cells of leukemic mice derived from the primary transplantation shown in Fig. 5B. The secondary recipients of *Inca1^−/−^*; MLL-AF9 cells (n = 15) died after a significantly longer latency than mice transplanted with *Inca1^+/+^*; MLL-AF9 primary blasts (n = 15; p<0.001). Secondary transplantation of *p16^−/−^*; MLL-AF9 blasts also had elongated survival compared to wild type-MLL-AF9 cells (p<0.001), but to a significant less extent (*Inca1^−/−^* vs *p16^−/^*−: p<0.001). **C.** Colony assays using MLL-AF9/GFP^+^c-kit^+^ *Inca1^+/+^* or *Inca1^−/−^* spleen blast, respectively. *Inca1^−/−^*; MLL-AF9 blasts formed less colonies *Inca1^+/+^* blasts (p = 0.05, t-test). **D.** For a cloning efficiency assay, *Inca1^+/+^*; MLL-AF9 or *Inca1^−/−^*; MLL-AF9 bone marrow cells from leukemia-transplanted mice were FACS-sorted and 1 to 300 c-kit^+^GFP^+^ cells were seeded in semi-solid medium in a 48-well plate. *Inca1^+/+^* cells had a clone forming frequency of 1/13, while the frequency was much lower in *Inca1^−/−^* cells (1/54; p = 0.0001). Shown here are the mean results of two independent experiments.

We next sought to confirm leukemia exhaustion independently in another model of murine AML. Transplantation of *Inca1*-deficient and wild type bone marrow retrovirally transduced with the oncogene MLL-AF9 [22], [23], [34] led to myeloid leukemia both in wild type and *Inca1^−/−^* bone marrow with comparable latency, penetrance, and morphology (Fig. 6A; p = 0.056). But *Inca1^−/−^*; MLL-AF9 leukemic cells generated a transplantable disease upon transplantation into secondary recipients with a significantly longer latency than *Inca1^+/+^*; MLL-AF9 cells (Fig. 6B; p<0.001), suggesting a hidden leukemia stem cell phenotype in the primary MLL-AF9-induced disease, which was uncovered upon the secondary transplantation. Remarkably, when we directly compared the effect of the absence of Inca1 to the absence of p16, a known tumor suppressor [35], [36], *Inca1^−/−^* MLL-AF9-leukemic blasts initiated a secondary leukemia significantly later than *p16^−/−^* blasts did (Fig. 6B).

As a third model of murine leukemia, we analysed the survival of c-myc/BCL2-transduced bone marrow cells, which led to comparable results as for MLL-AF9-transduced cells: No difference occurred between wild type and *Inca1*-knockout cells after primary transplantation (S2A Fig.) but the latency of leukemogenesis was significantly prolonged in absence of Inca1 after secondary transplantation (S2B Fig.).

In accordance with the effect of Inca1 in AML1-ETO9a-transduced bone marrow cells, FACS-isolated GFP^+^ c-kit^+^ MLL-AF9 blasts formed significantly fewer colonies in absence of Inca1 (Fig. 6C). Moreover, the frequency of colony forming units as determined in cloning efficiency assays was lower in GFP^+^ c-kit^+^ *Inca1^−/−^*; MLL-AF9 blasts than in wild type MLL-AF9 blasts (Fig. 6D).

Inca1 mRNA expression levels in AML blasts are lower than in normal bone marrow cells [15]. It is tempting to speculate that suppression of Inca1 permits expansion of the malignant clone but that a threshold of Inca1 is required to prevent leukemia exhaustion. Obviously, low Inca1 expression in patients and no Inca1 expression in genetically modified mice make a difference for leukemogenesis, as seen in AML1-ETO9a-positive cells. Therefore, it might be useful to further lower the INCA1 expression in leukemia patients to inhibit its function as cell cycle regulator and to reach an expression level near to absence that prohibits LSC maintenance. Since absence of Inca1 also does not appear to affect important organs and therefore survival, such a therapy approach might lack severe side effects as seen i.e. with FLT3-inhibitors. Here, FLT3 is essential for normal hematopoiesis [37], on the other hand mutated FLT3 triggers leukemogenesis in mice [38], [39] and man [40], [41]. Therefore, although FLT3-ITD seems to be a potent therapeutic target [41], [42], adverse reactions like incomplete hematological recovery after chemotherapy [43] could be anticipated according to the murine knockout phenotype [37]. Pharmacological inhibitors of the cell cycle have already become an attractive and effective therapy option (reviewed in [44]). Therapeutic targets like Inca1 that are at least not essential for survival of hematopoietic cells under homeostatic conditions might be a good choice to be tested.

In summary, we identify INCA1 as a novel regulator of leukemic stem cell function, which is largely dispensable for normal hematopoiesis under homeostatic conditions. The distinct functions in the regulation of stem/progenitor cells under physiological conditions and in leukemogenesis suggest that Inca1 might be a novel target for leukemia stem cell specific therapy approaches.

## Supporting Information

S1 Fig **Loss of Inca1 does not interfere with normal hematopoiesis.** **S1A.** FACS-analysis of bone marrow cells of *Inca1^+/+^* and *Inca1^−/−^* mice. The hematopoietic subpopulations were determined as Lin^−^ Sca-1^+^ c-Kit^+^ (LSK) cells, LT-HSC (CD34^−^/Flt3^−^), ST-HSC (CD34^+^/Flt3^−^) and MPPs (CD34^+^/Flt3^+^). Marked areas indicate Lin^−^ Sca-1^+^ c-Kit^+^. Results are summarized in 1B. **S1B.** The differentiation of colonies grown in methylcellulose did not differ between wild type and *Inca1^−/−^* cells. **S1C.** Colony assays using sorted LSK cells from *Inca1^+/+^* and *Inca1^−/−^* bone marrow. No differences could be observed between the two genotypes.
(EPS)

S2 Fig **Loss of Inca1 delays secondary transplanted c-myc/BCL2 leukemia.** **S2A.** Survival curves of recipient mice, which were transplanted with bone marrow cells of *Inca1^+/+^* or *Inca1^−/−^* mice retrovirally transduced with c-myc-IRES-BCL2 as depicted in Fig. 4A (n = 12 of each genotype). Cells of both genotypes led to a fatal leukemic disease with comparable latency and penetrance. **S2B.** Survival curves of secondary recipient mice which were transplanted with 10^6^ mCherry^+^ spleen cells of leukemic mice derived from the primary transplantation shown in Fig. S2A. The secondary recipients of *Inca1^−/−^*; c-myc/BCL2 cells (n = 11) died after a significantly longer latency than mice transplanted with *Inca1^+/+^*; c-myc/BCL2 primary blasts (n = 11; p = 0.005).
(EPS)
